# Associations between the phenotype and genotype of MnSOD and catalase in periodontal disease

**DOI:** 10.1186/s12903-019-0877-3

**Published:** 2019-08-30

**Authors:** Chang-Yu Lee, Chia-Huang Chang, Nai-Chia Teng, Hung-Ming Chang, Wan-Ting Huang, Yung-Kai Huang

**Affiliations:** 1 Division of Periodontics, Department of Dentistry, Taipei Medical University Hospital, Taipei Medical University, Taipei, 11031 Taiwan; 2 College of Public Health and Nutrition, Taipei Medical University, Taipei, 11031 Taiwan; 3 Department of Dentistry, Taipei Medical University Hospital, Taipei Medical University, Taipei, 11031 Taiwan; 4 Department of Anatomy and Cell Biology, School of Medicine, College of Medicine, Taipei Medical University, Taipei, 11031 Taiwan; 5 Department of Oral Hygiene, College of Dental Medicine, Kaohsiung Medical University, Kaohsiung, 80708 Taiwan; 6 School of Oral Hygiene, College of Oral Medicine, Taipei Medical University, Taipei, 11031 Taiwan

**Keywords:** Biomarker, Oxidative stress, Phenotype, Genetic polymorphism

## Abstract

**Background:**

Periodontal disease is an inflammatory disease in which pathogenic infections trigger a series of inflammatory responses and redox regulation. The hypothesis of this study was that a host’s redox regulation, as modified by genetic polymorphisms, may affect periodontal disease activities (including the plaque index (PlI), bleeding on probing (BOP), and pocket depth (PD)) during periodontal therapy.

**Methods:**

In total, 175 patients diagnosed with periodontitis were recruited from the Department of Periodontology, Taipei Medical University Hospital. Both saliva samples and clinical measurements (PlI, BOP, and PD) were taken at the baseline and at 1 month after completing treatment. Salivary manganese superoxide dismutase (MnSOD) and catalase, and corresponding genetic polymorphisms (*MnSOD*, T47C, rs4880 and *Catalase*, C-262 T, rs1001179) were determined. The extent of change (Δ) of MnSOD or catalase was calculated by subtracting the concentration after completing treatment from that at the baseline.

**Results:**

Subjects who carried the *Catalase* CC genotype had significantly higher salivary MnSOD or catalase levels. The *MnSOD* genotype had a significant effect on the percentage of PDs of 4~9 mm (*p* = 0.02), and salivary ΔMnSOD had a significant effect on the PlI (*p* = 0.03). The *Catalase* genotype had a significant effect on the PlI (*p* = 0.01~0.04), but the effect was not found for the mean PlI or PD. There was a significant interaction between the MnSOD genotype and salivary ΔMnSOD on PDs of 4~9 mm. After adjusting for gender, years of schooling, smoking status, and alcohol consumption, subjects with ΔMnSOD of < 0 μg/ml or Δcatalase of < 0 μg/ml had significantly higher 5.58- or 5.17-fold responses to scaling and root planing treatment.

**Conclusions:**

The *MnSOD* T47C genotype interferes with the phenotype of salivary antioxidant level, alters MnSOD levels, and influences the PD recovery. MnSOD and catalase gene polymorphism associated with phenotype expression and susceptibility in periodontal root planing treatment responses.

**Electronic supplementary material:**

The online version of this article (10.1186/s12903-019-0877-3) contains supplementary material, which is available to authorized users.

## Introduction

Periodontitis is an inflammatory disease that is initiated by the accumulation of plaque biofilm and its products, with subsequent gingival bleeding, alveolar bone resorption, and periodontal pocket formation [1]. Periodontal pathogenic infections trigger a series of inflammatory responses and lead to destruction of the periodontium [2]. Several lines of clinical evidence also indicated that cardiovascular diseases, diabetes mellitus, and other chronic diseases may contribute to periodontal inflammation, and the symptoms of systemic diseases can also be mitigated by preventing periodontal disease [3, 4].

The excessive production of reactive oxygen species (ROS) causes progressive oxidative damage via responses to periodontal injury and inflammation [5–7]. ROS, such as superoxide and hydroxyl species, are regulated by the thioredoxin system to transduce redox signals and alter activities of antioxidant enzymes to eliminate free radicals. Superoxide radicals (O_2_•-) are catalyzed into hydrogen peroxide (H_2_O_2_) by superoxide dismutase (SOD). H_2_O_2_ is then converted into H_2_O and O_2_ by catalase. In the decomposition of H_2_O_2_, hydroxyl radicals (OH•) formed by splitting O-O bonds can cause DNA and protein damage [8–10]. Salivary antioxidant activities, such as the total oxidant status, catalase, and SOD, have been useful biomarkers for evaluating the severity of periodontal disease and treatment effectiveness [11, 12].

Single-nucleotide polymorphisms (SNPs) contribute to expressions of genetic susceptibility to inflammatory and redox reactions in individuals with periodontitis [13]. The genotype (SNPs) and phenotype (gene expressions) of periodontal tissues can be used to evaluate susceptibility to periodontal disease and may also contribute to the effectiveness of clinical treatments.

A polymorphism of the manganese SOD (MnSOD) gene (T47C, rs4880) affects the redox status balance through altering enzyme localization and mitochondrial transportation, and the MnSOD T47C SNP is also controlled by environmental factors [14]. A polymorphism of the *Catalase* gene (C-262 T, rs1001179) is located in the promoter region, and it has a functional impact on catalase expression [15]. Activities of MnSOD and catalase differ due to allelic frequencies which account for ethnic variations. Frequencies of *MnSOD* T47 and *Catalase* C-262 respectively range 23%~ 29 and 61%~ 69% in Caucasians and 66%~ 75 and 90%~ 93% in Asians [16–19]. Both of these polymorphic variants can alter enzymatic activities against oxidative damage and modulate individual susceptibility to disease occurrence. It was also shown that genetic polymorphisms of these antioxidants were associated with promoting antioxidative effects against the risks of cancer and tumorigenesis [18, 19].

The goal of periodontal treatment is to recover periodontal health and function, maintain esthetics of the dentition, and achieve effective infection control [20]. However, limited treatment effectiveness related to redox homeostasis and individual susceptibility has seldom been reported. The hypothesis is that a host’s inflammatory response, as modified by genetic polymorphisms and salivary antioxidant levels, can affect the effectiveness of periodontal treatment. The objective of this study was to explore associations of genetic polymorphisms and salivary expressions of MnSOD and catalase with the effectiveness of periodontal disease treatment.

## Materials and methods

### Subject recruitment

Participants were enrolled from the Division of Prosthodontics, Department of Dentistry at Taipei Medical University (TMU) Hospital between July 2013 and April 2016. Subjects who were eligible for a comprehensive periodontal treatment project (CTPT) were recruited. The CTPT is a National Health Insurance program to reduce periodontal disease in Taiwan [21]. Subjects who met all of the following criteria were included in this study: the patient had been diagnosed with ICD-9523, this was their first visit for periodontitis treatment, the number of functional teeth was > 15, the probing depth was ≥5 mm for at least six teeth, and the patient had not been treated with non-surgical therapy. Patients who had received periodontal therapy, were pregnant, or had been diagnosed with cancer were excluded from the study. This study was approved by the Research Ethics Committee of the TMU Joint Institutional Review Board (Taipei, Taiwan), and complied with the World Medical Association *Declaration of Helsinki*.

All participants provided written informed consent before the questionnaire interview and salivary specimen collection. A previous epidemiological study showed that factors such as age, gender, educational level, and tobacco and alcohol use were risk factors for periodontal disease [3]. Before periodontal treatment, each participant completed a structured questionnaire that collected sociodemographic characteristics (gender and years of schooling), lifestyle factors (cigarette smoking, alcohol consumption, and betel nut chewing), personal and family disease histories, and oral hygiene knowledge, attitudes, and behaviors. According previous study [22], the smoking status was defined as “current smokers” (who smoked more than 100 cigarettes in his or her lifetime and currently smokes cigarettes), “former smokers” (who have smoked more than 100 cigarettes in the pasts and currently not smoking), and “never smokers” (who had never smoked in their lifetime). Alcohol consumption was defined as current (drinks alcoholic beverages more than 3 times per week), former (stopped drinking alcoholic beverages for ≥1 year or has occasionally drunk alcoholic beverages in their lifetime), and never (has not drunk alcoholic beverages in their lifetime).

**Fig. 1:**
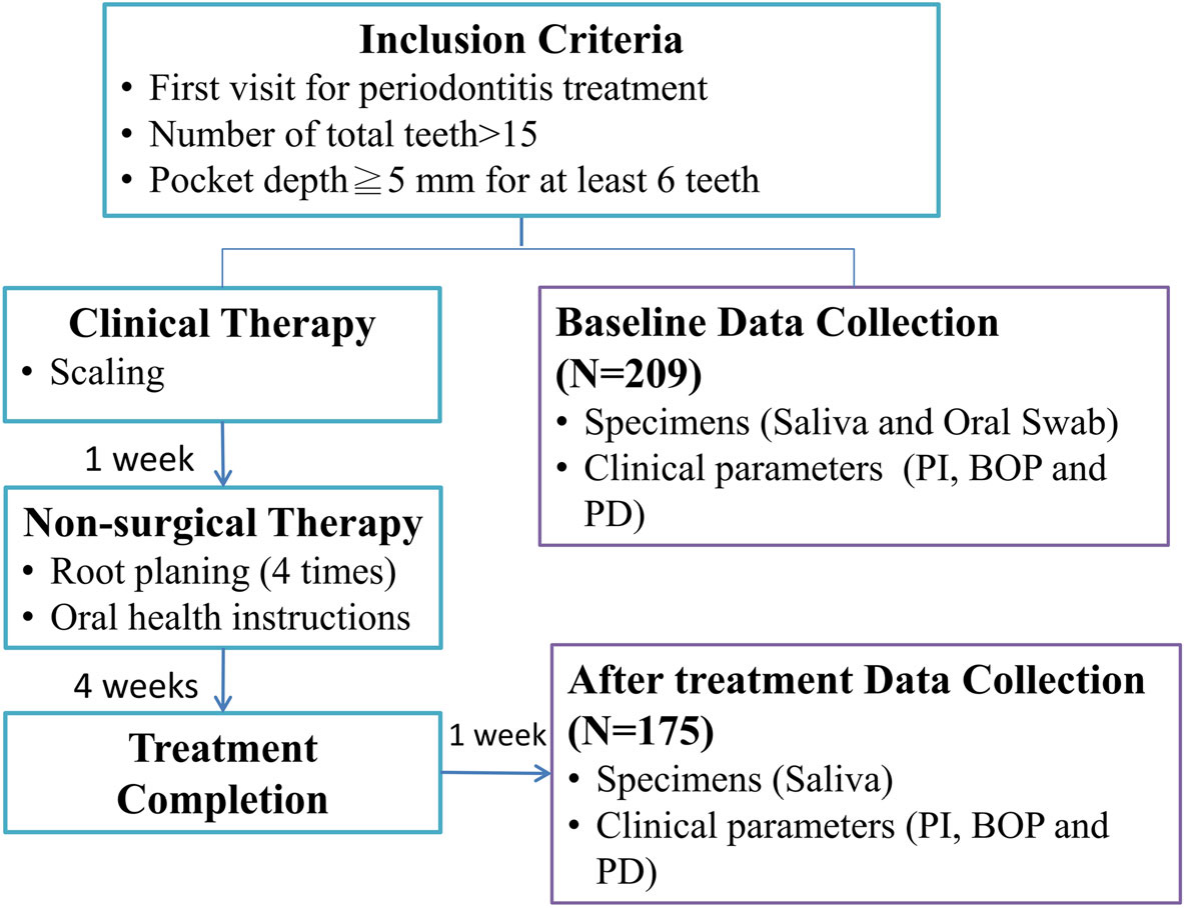
Flowchart of participant enrollment

Figure 1 is a flowchart of participant enrollment. After patients had completed the informed consent form, 209 patients were invited to participate in this study by convenience sampling. The structured questionnaire included demographics, socioeconomic status, cigarette smoking status (quantity, duration, and pack-years), alcohol consumption, and frequency of betel nut chewing and was carried out by well-trained interviewers. A previous study indicated that it is better to treat periodontitis for a period of time (e.g., once a week continuously for 1 month, as demonstrated in our current study). The advantages of this strategy may include: (1) patients feel less stressful during the stable therapeutic process; (2) oral hygiene can be further improved by increasing the number of treatments; and (3) patients can even establish or enhance their reliance on these regular therapeutic procedures [23]. In the present study, all patients completed the therapeutic process within 4 weeks. The therapeutic efficacy of periodontitis was evaluated 1 week after treatment by measuring data collected from healing samples. After excluding those with incomplete data on clinical parameters or genotype, there were 175 patients who completed scaling and root planing.

The sample size estimation was based on the paper published by Novakovic et al. [24]. The value of the means of salivary SOD before and after the treatment of scaling and root planing were counted to be 0.45 and 0.39, respectively. G*Power software (version 3.1.9.4) and program of “Means: Difference between 2 dependent means (matched/paired samples t-test)” was used to calculate the sample size [25]. The correlation of salivary SOD before and after the treatment was not showed in the previous study, so here we assumed the correlation as 0.1 and 0.5, respectively. Under the correlation of 0.1 and 0.5, the effect size from the mean difference (0.06) was 0.24 and 0.30, respectively. With the statistical power of 0.8, alpha of 0.05, two tails with an effect size of 0.30 and 0.24, the required sample size was therefore calculated to be 89 to 136 pairs, respectively.

### Specimen collection

Saliva samples were collected at the baseline (before the non-surgical intervention) and at 1 week after completing clinical treatment. Participants were asked to chew a piece of wax for 5 min to collect saliva using the Saliva-Check kit (GC Corporation, Tokyo, Japan). Oral swab specimens were also collected. Subjects rinsed their mouth with water to remove food residue and waited at least 10 min after rinsing to avoid specimen dilution before saliva collection. After collection, saliva and oral swab samples were stored in an ice bucket and immediately transported to the laboratory. Saliva samples were mixed with a protease inhibitor cocktail (Roche Applied Science, Mannheim, Germany) at a ratio of 1 ml saliva: 10 μL protease inhibitor cocktail and centrifuged (3000 rpm) for 3 min at room temperature. Supernatants were collected and stored at − 20 °C until analysis. Saliva samples were analyzed for oxidative stress biomarkers.

### Salivary antioxidants determination

MnSOD and catalase levels were determined by an immunoassay using the MILLIPLEX® MAP Human Oxidative Stress Magnetic Bead Panel kit (Merck Millipore, Darmstadt, Germany). Each sample (35 μL) diluted to an identical quantity of protein with assay buffer was added to a 96-well plate, mixed with 35 μL of assay buffer and 25 μL of antibody-immobilized beads, and incubated 2 h at room temperature, followed by the addition of detection antibodies (50 μL) and streptavidin phycoerythrin (50 μL) incubation. The mean fluorescence intensity (MFI) was determined. The *R*^2^ value for the standard curve was > 0.995. Coefficients of variance (CVs) for the intra-assay ranged 3.49%~ 8.10%, and CVs for the inter-assay ranged 1.22%~ 12.30%. A laboratory negative control was not included in the manufacturer’s instructions of MILLIPLEX® MAP Human Oxidative Stress Magnetic Bead Panel kit. The extent of change (Δ) of MnSOD or catalase was calculated by subtracting the concentration after completing treatment from that at the baseline.

### Determination of *MnSOD* and *catalase* genetic polymorphisms

Genomic DNA was extracted from mouth swabs by a QIAamp DNA Investigator Kit in accordance with the manufacturer’s instruction (Qiagen, Hilden, Germany). *MnSOD* T47C and *Catalase* C-262 T were genotyped by a polymerase chain reaction-restriction fragment length polymorphism (PCR-RFLP) method modified from earlier studies [15, 26]. Genetic polymorphisms of *MnSOD* (a substitution of the T47C polymorphic site located on chromosome 6 q 25) and *Catalase* (a substitution of the C-262 T polymorphic site located on chromosome 11 p 13) were determined by the PCR-RFLP method. MnSOD primers were 5′-GCACCAGCAGGCAGCTGGCGCCGG-3′ and 5′-TGCGCGTTGATGTGAGGTTCCAG-3′. *Catalase* primers were 5′-AGAGCCTCGCCCCGCCGGACCG-3′ and 5′-TGCGCGTTGATGTGAGGTTCCAG-3′. Initial denaturation was set to 94 °C for 5 min, followed by 35 cycles at 94 °C for 30 s, 57 °C for 30 s, and 72 °C for 30 s. A final extension was prolonged for 5 min. DNA fragments were amplified with restriction endonucleases, visualized through 3% agarose gel electrophoresis, stained, and photographed under UV light. Wild-type (TT) MnSOD was characterized as a 112-bp fragment, while the mutant types (TC and CC) were 90- and 22-bp fragments, respectively. Two fragments of 155 and 30 bp were characterized as the wild-type (CC) of *Catalase* and a 185-bp fragment as the mutant type (CT or TT). The validity of genotyping was determined by the Hardy-Weinberg Law and DNA sequencing. Around 25% of the samples were genotyped in duplicate for these two SNPs, and the concordance rate was 100%.

A previous study showed that subjects carrying the less-common T allele (CT and TT) of *catalase* had significantly higher catalase activity compared to that of CC homozygotes [15]. Sutton et al. demonstrated that the less-common C allele (TC and TT) of *MnSOD* had significantly higher messenger (m)RNA expression compared to TT homozygotes [27]. The less-common allele of these two genes was related to higher expression. According to the function of the genotype, genotypes of *MnSOD* were classified as TT and TC/CC, and those of catalase were classified as CC and CT/TT.

### Clinical parameters and treatment evaluation

Clinical examinations and non-surgical periodontal treatments, such as subgingival scaling, root planing, and oral hygiene instructions, were carried out. In order to reduce inter- and intra-variations of clinical parametric assessments, all clinical parametric assessments were performed by the same periodontist. The periodontist followed examiner alignment and assessment in periodontal research published by Hefti and Preshaw [28], to perform all clinical parametric assessments in this study.

Measurement of the plaque index (PlI) was based on both soft debris and mineralized deposits on four surfaces (buccal, lingual, mesial, and distal) of a tooth, and the presence or absence of plaque was recorded at all sites. The PlI was calculated by dividing the number of plaque-containing surfaces by the total number of available surfaces [29]. The bleeding on probing (BOP) and pocket depth (PD) were measured using a periodontal probe (Color Coded Michigan Williams Dental Probe) at six sites (distobuccal, buccal, mesiobuccal, distolingual, lingual, and mesiolingual) on each tooth. The BOP and PD were expressed as a percentage, which was calculated by dividing the number of bleeding sites or sites with PDs by the total number of available sites. The average periodontal PD (PD mean) was also calculated as an index of clinical parameters.

Studies have thus far not identified a level of plaque infection compatible with maintenance of periodontal health. However, in a clinical setup, a plaque control record of 20%~ 40% might be tolerated by most patients. It is important to realize that the full-mouth plaque score has to be related to the host response of the patient, in other words compared to inflammatory parameters [30]. According to a previous study, periodontal pockets deeper than 4.2 mm were associated with periodontal attachment gain after periodontal surgery [31]. As modified from two previous studies, participants were classified into responsive and non-responsive groups according to the clinical indices. The non-responsive group was defined if the PlI exceeded 30% and PD mean exceeded 3 mm after treatment. The opposite defined the responsive group.

### Statistical analysis

**Fig. 2:**
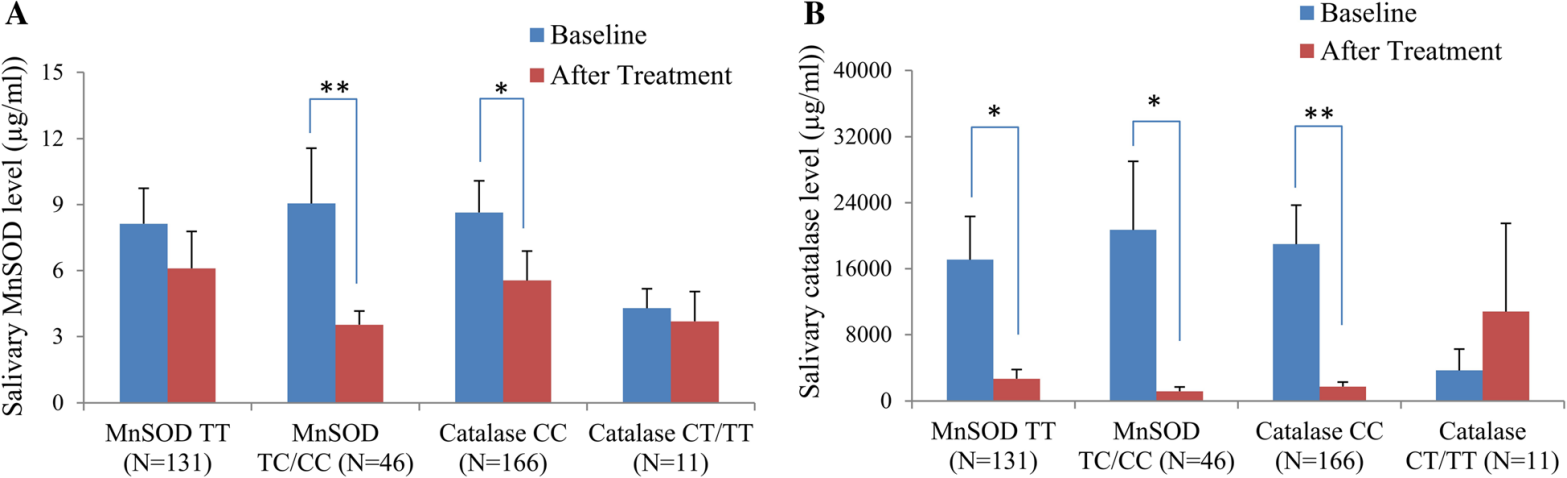
Salivary antioxidant biomarker levels at the baseline and after treatment completion. The histogram with the error bar displays the mean and standard error. **a** Manganese superoxide dismutase (MnSOD); **b** Catalase. * *p* < 0.05 and ** *p* < 0.01, according to a paired *t*-test

The SAS program vers. 9.4 (SAS Institute, Cary, NC, USA) was used for all statistical analyses. A multiple general linear regression analysis was used to determine contributions of demographic characteristics (independent variables) to periodontal clinical parameters (dependent variables) (Table 1). Independent variables in Table 1 were recorded as follows: gender (male was recorded as (1), female was recorded as (0)), years of schooling (> 12 years was recorded as (1), ≤12 years was recorded as (0)), smoking status (current and former smoker was recorded as (1), never having smoked was recorded as (0)), and alcohol consumption (current and former consumer was recorded as (1), never consumed was recorded as (0)). Differences in periodontal clinical parameters, in salivary antioxidant levels, and among the genotypes of antioxidants were determined using Student‘s *t*-test (Table 2). Differences in salivary antioxidant levels at the baseline and after treatment were evaluated with a paired *t*-test (Fig. 2).

**Table 1:** Multiple general linear regressions of demographic characteristics on periodontitis clinical parameters in patients with periodontal disease

	Plaque index (%)		Bleeding on probing (%)		Percentage of PDs of 4~9 mm (%)		Mean PD (mm)	
	Baseline	After treatment	Baseline	After treatment	Baseline	After treatment	Baseline	After treatment
	β (SE)		β (SE)		β (SE)		β (SE)	
Gender								
Male vs. Female	−0.21 (3.40)	3.44 (2.98)	5.55 (3.78)	3.46 (2.92)	2.35 (2.64)	1.00 (1.73)	0.06 (0.09)	0.04 (0.06)
Years of schooling								
> 12 vs. ≤12	0.67 (2.88)	−0.15 (2.52)	−8.66 (3.20)**	−2.97 (2.47)	−4.43 (2.23)*	−1.46 (1.46)	−0.15 (0.08)	− 0.03 (0.05)
Smoking status								
Current and quit vs. Never	−3.16 (3.70)	−2.73 (3.24)	−9.55 (4.12)*	−1.00 (3.18)	1.57 (2.87)	3.35 (1.88)	0.01 (0.10)	0.11 (0.07)
Alcohol consumption								
Current and quit vs. Never	4.09 (3.54)	−0.18 (3.10)	0.42 (0.91)	−0.68 (3.04)	−1.11 (2.75)	−1.47 (1.80)	0.01 (0.09)	−0.05 (0.06)

*p < 0.05 and **p < 0.01.

*SE* standard error, *PD* pocket depth

**Table 2:** Distribution of periodontitis clinical parameters and salivary antioxidant levels among subgroups of *MnSOD* and *Catalase* genotypes

Baseline	*MnSOD* genotype		*p* value for *t*-test	*Catalase* genotype		*p* valuefor *t*-test
	TT (*N* = 129)	TC/CC (*N* = 46)		CC (*N* = 164)	CT/TT (*N* = 11)	
	Mean ± SE			Mean ± SE		
PlI (%)	58.67 ± 1.65	61.56 ± 2.72	0.36	60.09 ± 1.45	49.64 ± 5.59	0.07
BOP (%)	43.24 ± 1.81	44.51 ± 3.47	0.72	43.48 ± 1.68	44.96 ± 5.89	0.82
PD of 4~9 mm (%)	29.96 ± 1.28	32.5 ± 2.22	0.31	30.36 ± 1.13	34.67 ± 5.18	0.34
PD mean	3.45 ± 0.05	3.47 ± 0.0.7	0.88	3.45 ± 0.04	3.60 ± 0.17	0.37
MnSOD (μg/ml)	7.04 ± 1.17	9.05 ± 2.51	0.46	7.79 ± 1.16	4.29 ± 0.88	0.02
Catalase (μg/ml)	17,347.67 ± 5322.96	20,703.85 ± 8324.45	0.74	19,203.72 ± 4771.04	3710.52 ± 2532.08	< 0.01
After treatment						
PlI (%)	37.33 ± 1.44	36.12 ± 2.44	0.66	36.61 ± 1.24	43.04 ± 6.76	0.20
BOP (%)	21.7 ± 1.27	25.16 ± 2.95	0.28	22.07 ± 1.21	30.76 ± 6.86	0.23
PD of 4~9 mm (%)	12.99 ± 0.84	12.7 ± 1.47	0.86	12.81 ± 0.75	14.47 ± 3.3	0.57
PD mean	2.79 ± 0.03	2.80 ± 0.05	0.88	2.79 ± 0.03	2.87 ± 0.12	0.46
MnSOD (μg/ml)	4.95 ± 1.4	3.53 ± 0.62	0.35	4.64 ± 1.11	3.69 ± 1.36	0.59
Catalase (μg/ml)	2729.82 ± 1130.27	1167.21 ± 525.66	0.21	1750.44 ± 557.49	10,796.95 ± 10,701.48	0.41
Difference						
Δ MnSOD (μg/ml)	2.09 ± 0.53	5.52 ± 1.95	0.09	3.15 ± 0.69	0.6 ± 0.62	< 0.01
ΔCatalase (μg/ml)	14,617.85 ± 5200.55	19,536.63 ± 8085.25	0.62	17,453.29 ± 4611.44	− 7086.43 ± 8551.82	0.02

*MnSOD* manganese superoxide dismutase, *PlI* plaque index, *BOP* bleeding on probing, *PD* pocket depth, *SE* standard error

To further explore interactions of salivary antioxidant levels and genotypes with periodontal clinical parameters, a two-way repeated-measure analysis of variance (ANOVA) was performed to compare subject performance according to the clinical parameters before and after treatment, using differences in genotypes and salivary antioxidant levels as the main effects (independent variables). The effect of genotype was classified into two levels (TT and TC/CC for *MnSOD* and CC CT/TT for *Catalase*). The effect on differences in salivary antioxidant levels was a continuous variable.

Univariate and multiple logistic regressions were used to estimate the odds ratios (ORs) of scaling root planing responses to genotypes and phenotypes of antioxidants (Table 4). The effects of salivary ΔMnSOD and Δcatalase were classified into two levels (≥0 and < 0 μg/ml), If the change of the antioxidants was less than 0 μg/ml, it means that the level of the antioxidants was increased during treatment. Nevertheless, if the change of the antioxidants was greater than 0 μg/ml, than it means that the level of the antioxidants was decreased during treatment. The adjusted potential confounders as independent variables (gender, years of schooling, smoking status, and alcohol consumption) in the multiple logistic regression models were the same as those in Table 1. The level of significance was set to *p* < 0.05 for all statistical tests.

## Results

Among 175 participants, consisting of 80 males and 95 females, the average age was 55.55 years. More than half (57.06%) of participants had a bachelor’s degree. The baseline BOP percentage and PDs of 4~9 mm in subjects who had > 12 school years were significantly lower than those of subjects who had ≤9 years of school (*p* < 0.01 and *p* = 0.02). The majority of subjects did not smoke or drink alcohol. There were no significant differences in percentages of the baseline/after treatment for the PlI, BOP, or PD in groups stratified be sex, smoking status, and alcohol consumption (Additional file 1: Table S1). Table 1 shows results of multiple general linear regressions of demographic characteristics on periodontitis clinical parameters in patients with periodontal disease. No demographic characteristics were associated with the baseline PlI or PD mean. After adjusting for gender, smoking status, and alcohol consumption, baseline percentages of BOP and PD of 4~9 mm were significantly associated with years of schooling. Compared to subjects who had ≤12 years of schooling, subjects who had > 12 years of schooling had significantly lower baseline percentages of BOP (8.66%, *p* < 0.01) and PD of 4~9 mm (4.43, *p* < 0.05). The regression coefficients of the baseline BOP decreased after adjusting for gender, smoking status, and alcohol consumption. The baseline BOP in current smokers and those who had quit smoking was significantly 9.55% lower than that of non-smokers (*p* < 0.05). No demographic characteristics were associated with any periodontal clinical parameters after treatment.

Table 2 shows distributions of periodontitis clinical parameters and salivary MnSOD and catalase levels among the subgroups of *MnSOD* and *Catalase* genotypes. There were no significant differences in baseline clinical parameters between *MnSOD* genotype strata. Subjects who carried the *Catalase* CC genotype had significant higher salivary levels of MnSOD and catalase than did subjects who carried the *Catalase* CT/TT genotype (*p* = 0.02 and *p* < 0.01, respectively). ΔMnSOD and ΔCatalase were significant higher in subjects who carried the *Catalase* CC genotype than those with the *Catalase* CT/TT genotype (*p* < 0.01 and *p* = 0.02, respectively).

Figure 2 shows differences in salivary antioxidant levels at the baseline and after treatment completion. A significant reduction in the MnSOD level after treatment was found in subjects who carried the *MnSOD* TT, *MnSOD* TC/CC, or *Catalase* CC genotype (Fig. 2a). A significant reduction in the catalase level after treatment was found in subjects who carried the *MnSOD* TT, *MnSOD* TC/CC, or *Catalase* CC genotype (Fig. 2b).

A repeated-measures ANOVA was used to calculate the adjusted percentage changes in PlI, BOP, and PD of 4~9 mm for the combined effect of genotype and differences in salivary oxidative levels (Table 3). Salivary ΔMnSOD had a significant effect on the PlI (*p* = 0.03). When adjusted for the *MnSOD* genotype, PlI changes decreased by 0.43% with an increase of 1 μg/ml in ΔMnSOD. When adjusted for the ΔMnSOD level, the *MnSOD* genotype had a significant effect on the percentage of PDs of 4~9 mm (*p* = 0.02). The decrease in the percentage of PDs of 4~9 mm in subjects who carried the *MnSOD CC* genotype was 2.82% significantly lower than those in subjects with the *MnSOD CT/TT* genotype (16.97% vs. 19.79%). There was a significant interaction between the *MnSOD* genotype and ΔMnSOD in those with PDs of 4~9 mm (*p* < 0.01), when adjusting for the ΔMnSOD level, as that of subjects who carried the *MnSOD CC* genotypes was 4.23% significantly lower than that in subjects with the *MnSOD CT/TT* genotype. There was no significant interaction between the *MnSOD* genotype and Δcatalase in terms of clinical parameters. When adjusting for the ΔMnSOD level or Δcatalase level, the *Catalase* genotype had a significant effect on the PlI. When adjusting for the ΔMnSOD level or Δcatalase level, the decrease in the percentage of PlI in subjects who carried the *Catalase CC* genotype was 13.84% (*p* = 0.04) or 17.70% (*p* = 0.01) significantly higher than those of subjects who carried the *Catalase CT/TT* genotype, respectively. The effect was not found in the mean change in percentage of PlI or PDs of 4~9 mm between *Catalase CC* genotypes and the salivary ΔMnSOD level or Δcatalase level. Finally, there was no significant interaction between the *Catalase* genotype and ΔMnSOD or Δcatalase on clinical parameters.

**Table 3:** Results of two-way repeated-measures ANOVA comparing the main effects of genotype and salivary antioxidant levels on clinical parameters

	Dependent variable					
	PlI (%)		BOP (%)		PDs of 4~9 mm mean (%)	
	*F* value	*p* value	*F* value	*p* value	*F* value	*p* value
Independent variable						
*MnSOD* T47C genotype effect	0.48	0.48	0.29	0.59	5.71	0.02
Salivary ΔMnSOD effect	4.39	0.03	3.36	0.07	3.46	0.06
*MnSOD* T47C × ΔMnSOD effect	0.00	0.94	1.03	0.31	8.61	<0.01
*MnSOD* T47C genotype effect	1.86	0.17	0.07	0.79	3.20	0.08
Salivary Δcatalase effect	0.51	0.47	1.96	0.16	0.01	0.93
*MnSOD* T47C × Δcatalase effect	1.39	0.24	2.16	0.14	0.49	0.48
*Catalase* C-262T genotype effect	4.04	0.04	0.34	0.56	1.43	0.23
Salivary ΔMnSOD effect	0.57	0.45	2.12	0.14	0.58	0.44
*Catalase* C-262T × ΔMnSOD effect	1.02	0.31	2.66	0.11	0.88	0.35
*Catalase* C-262T genotype effect	6.67	0.01	1.56	0.21	0.32	0.57
Salivary Δcatalase effect	0.57	0.45	0.60	0.44	1.55	0.21
*Catalase* C-262T ×Δcatalase effect	1.25	0.26	1.92	0.16	2.09	0.15

*MnSOD* manganese superoxide dismutase, *PlI* plaque index, *BOP* bleeding on probing, *PD* pocket depth

Table 4 shows ORs of scaling and root planing treatment response in genotypes and phenotypes of antioxidants. In the univariate logistic regression model, a significant association was observed between salivary antioxidant changes and responses to scaling and root planing treatments. Compared to subjects who had ΔMnSOD of ≥0 μg/ml, subjects who had ΔMnSOD of < 0 μg/ml had a significantly higher 4.86-fold response to scaling and root planing treatment. Compared to subjects who had Δcatalase of ≥0 μg/ml, subjects who had Δcatalase of < 0 μg/ml had a significantly higher 5.03-fold response to scaling and root planing treatment. After adjusting for gender, years of schooling, smoking status, and alcohol consumption, subjects with ΔMnSOD of < 0 μg/ml or Δcatalase of < 0 μg/ml had a significantly higher 5.58- or 5.17-fold response to scaling and root planing treatment. Salivary antioxidants of subjects increased during treatment to increase the treatment response.

**Table 4:** Odds ratios (ORs) of scaling and root planning treatment response in genotypes and phenotypes of antioxidants

	Non-response *N* (%)	Response *N* (%)	Crude model OR (95% CI)	Model I^a^	Model II^a^
				Adjusted OR (95% CI)	
*MnSOD* genotype					
TT	20 (71.43)	109 (74.15)	1.00	1.00	
CT/CC	8 (28.57)	38 (25.85)	0.87 (0.35~2.14)	1.08 (0.41~2.85)	
*Catalase* genotype					
CC	23 (92.86)	138 (93.88)	1.00		1.00
CT/TT	2 (7.14)	9 (6.12)	0.84 (0.17~4.15)		0.99 (0.17~5.56)
ΔMnSOD (μg/ml)					
≥ 0	26 (92.86)	107 (72.79)	1.00	1.00	
< 0	2 (7.14)	40 (27.21)	4.86 (1.10~21.42)*	5.58 (1.22~25.49)*	
ΔCatalase (μg/ml)					
≥ 0	26 (92.86)	106 (72.11)	1.00		1.00
< 0	2 (7.14)	41 (27.89)	5.03 (1.14~22.15)*		5.17 (1.15~23.21)*

^a^Adjusted for gender, years of schooling, smoking status and alcohol consumption

*p<0.05

*MnSOD* manganese superoxide dismutase, *CI* confidence interval

## Discussion

In this study, salivary MnSOD and catalase were significantly reduced after treatment, and an interaction between the genotype and phenotype of MnSOD was observed in the PD treatment effect. Saliva that contains unique information on oral physiological changes can be a useful diagnostic tool for periodontal diseases. Specific oxidative stress biomarkers, such as lipid peroxidation levels, the total oxidant status, and antioxidant levels, can reflect the severity of periodontal disease and treatment effectiveness [32–35]. A recent study mentioned that SOD was correlated with inflammatory diseases and could reflect the onset of disease [36]. An increase in antioxidant activity was accompanied by an early inflammatory syndrome, while its alleviation occurred in response to pathological progression. Novakovic et al. found that patients with periodontitis had higher antioxidant levels compared to periodontally healthy subjects, and antioxidant levels were significantly alleviated after non-surgical treatment [24]. Our previous study also demonstrated that an increase in SOD was related to the severity of periodontitis and to oral health behaviors [37]. Results of this study were consistent with those previous studies mentioned above; salivary MnSOD was associated with PlI changes. The importance of salivary antioxidants as prognostic biomarkers of periodontal treatment should be addressed.

Polymorphisms of antioxidants can modulate genetic activity and formation of antioxidants. In vitro, the alanine allele (*MnSOD* TC/CC) increased the activity of the MnSOD homotetramer and produced more-efficient import of MnSOD into the mitochondrial matrix compared to the valine allele (*MnSOD* TT) [38]. The high transcriptional activity of *Catalase* T variants was determined in HepG2 and K562 cells. Individuals who carried the *Catalase* T allele had higher catalase levels compared to those who carried the C allele [15]. Nevertheless, there were no significant differences in MnSOD or catalase activities regardless of the genotype in this study. Hong et al. also found similar results [39]. In addition, genetic polymorphisms have been associated with the susceptibility to disease occurrence and development. Kakkoura et al. indicated that wild-type alleles of *MnSOD* and *Catalase* SNPs may promote antioxidative effects of the Mediterranean diet against breast cancer risk [19]. In this study, the significant association between genetic variations and periodontal treatment existed in the *MnSOD* and *Catalase* genotypes, but there was no interaction between genotype and phenotype. Further studies are needed to explore relationships among genetic polymorphisms, enzymatic activities, and therapeutic responses.

Oral health is recognized as an essential and integral component of one’s general health and well-being; a major concern is the high prevalence of periodontal disease worldwide [40, 41]. The United States National Health and Nutrition Examination Survey (NHANES) showed that the periodontal disease prevalence in adults aged ≥30 years had decreased from 47.2% (NHANES 2009–2010) to 44.8% (NHANES 2011–2012) [42, 43]. In Taiwan, it was estimated that approximately 54% of adults aged 35~44 years have mild to severe periodontitis [44]. The prevalence of periodontal disease has significantly increased in Taiwan over the past 17 years [45]. Compared to other countries, such as India (72%), Italy (35%~ 40%), and the US (46%), it is noteworthy that there is a higher prevalence of periodontitis in Taiwan [46–48].

Periodontal disease results from periodontal pathogenic infections that induce a series of inflammatory and redox responses that lead to destruction of periodontal tissues and even tooth loss [7]. Non-surgical periodontal treatments, such as scaling and root planing, are the primary and initial steps for cleaning root surfaces and removing plaque and calculus from deep periodontal pockets; these are simultaneously used in coordination with oral hygiene instructions and ongoing maintenance of oral health behaviors [37]. The goals of periodontal treatment are to recover the periodontal health and function, maintain esthetics of the dentition, and achieve effective infection control and periodontal tissue regeneration [20]. In general, the majority of participants exhibit good responses to non-surgical periodontal treatments. Compared to other studies in terms of periodontal disease activities, reductions in the PlI, BOP, and PD in this study were clinically acceptable [49–51]. However, around 10% of patients still had increased PlI, BOP, and PD after treatment, and the results showed that oxidative stress played an important role during periodontal treatment.

Smoking is an important environmental risk factor for periodontal disease. Free radicals generated by cigarette smoke affect antioxidant systems in the body [7, 24, 52]. Cigarette smoke may interfere with inflammatory defense mechanisms of periodontal tissues and inhibit functions against plaque bacteria, thus reducing the effectiveness of periodontal treatment [53]. In the US NHANES, 41.9% of adult periodontitis cases were attributable to current cigarette smoking and 10.9% to former smoking [54]. Previous studies indicated that smokers exhibit less improvement than nonsmokers following non-surgical periodontal treatment [49, 55]. Preshaw et al. indicated that non-smokers tend to have less-advanced periodontitis at the baseline and better responses to non-surgical periodontal treatment [56]. Smokers had a higher percentage of PDs of 4~9 mm in this study.

Effective plaque control is the most important step in preventing dental caries and periodontal diseases [57–60]. Risk factors for periodontal disease, including age, gender, education, an unhealthy diet, tobacco use, alcohol use, dental care, etc., have been studied in epidemiological research [3, 61]. In spite of significant associations among antioxidants, genetic polymorphisms, and periodontal treatments, there is no denying that patients with poor responses to treatment have inferior health statuses, and this may have biased the results. In addition, the small sample size derived from low allelic frequency is a major limitation of this study. The less-frequent variant of the *catalase* gene in this study was similar to that in other Asian countries, such as Korea and China [62, 63]. Other factors, including dietary intake, the nutritional status, and dental care, that were not accurately measured in this study need to be further accounted for in future studies.

## Conclusions

The *MnSOD* T47C genotype interferes with the phenotype of salivary antioxidant level, alters MnSOD levels, and influences the recovery percentage of PDs of 4~9 mm. MnSOD and catalase gene polymorphism associated with phenotype expression and susceptibility in periodontal root planing treatment responses.

## Additional file

Additional file 1: **Table S1.** Demographic characteristics and periodontitis clinical parameter of patients. (DOC 70 kb)

## Data Availability

The dataset used during the study are available from the corresponding author upon request.

## Abbreviations

ANOVA: Analysis of variance
BOP: Bleeding on probing
CTPT: Comprehensive periodontal treatment project
MnSOD: Manganese superoxide dismutase
PD: Pocket depth
PlI: Plaque index
ROS: Reactive oxygen species
SNP: Single-nucleotide polymorphism
SOD: Superoxide dismutase
