# Supplementary material for: Associations between the phenotype and genotype of MnSOD and catalase in periodontal disease
Source: BMC Oral Health. 2019 Aug 30;19:201. doi: 10.1186/s12903-019-0877-3 (PMC6717336; doi:10.1186/s12903-019-0877-3)
Supplement: Supplementary file 1 — Table S1. Demographic characteristics and periodontitis clinical parameter of patients. (DOC 70 kb) [file 12903_2019_877_MOESM1_ESM.doc]

Additional file 1

Table S1. Demographic characteristics and periodontitis clinical parameter of patients

|  | *N* (%) | Plaque index (%) | | Bleeding on probing (%) | | Pocket depth of 4~9 mm (%) | |
| --- | --- | --- | --- | --- | --- | --- | --- |
|  |  | Baseline | After treatment | Baseline | After treatment | Baseline | After treatment |
|  |  | Mean±SE | | Mean±SE | | Mean±SE | |
| Overall | 175 | 59.43±1.41 | 37.01±1.23 | 43.57±1.61 | 22.61±1.22 | 30.63±1.11 | 12.91±0.73 |
| Gender |  |  |  |  |  |  |  |
| Female | 95 (54.29) | 59.46±1.83 | 36.02±1.73 | 43.01±2.12 | 21.39±1.43 | 29.48±1.35 | 12.04±0.89 |
| Male | 80 (45.71) | 59.39±2.2 | 38.19±1.75 | 44.24±2.49 | 24.07±2.04 | 31.99±1.81 | 13.94±1.18 |
| *p* value ^a^ |  | 0.98 | 0.38 | 0.70 | 0.28 | 0.26 | 0.19 |
| Years of schooling | | | | | | | |
| ≤9 years | 15 (8.57) | 56.05±4.11 | 29.99±3.79 | 58.97±6.87^#^ | 28.34±5.99 | 39.16±5.32^#^ | 14.47±3.28 |
| 10~12 years | 60 (34.29) | 59.77±2.39 | 38.68±2.27 | 45.4±2.28 | 23.17±1.98 | 31.65±1.72 | 13.65±1.37 |
| >12 years | 100 (57.14) | 59.74±1.92 | 37.07±1.56 | 40.17±2.15^#^ | 21.42±1.52 | 28.73±1.40^#^ | 12.23±0.85 |
| *p* value ^b^ |  | 0.76 | 0.18 | <0.01 | 0.28 | 0.02 | 0.53 |
| Smoking |  |  |  |  |  |  |  |
| Never | 125 (71.43) | 59.93±1.63 | 37.29±1.49 | 45.23±1.92 | 22.38±1.46 | 29.85±1.3 | 11.93±0.78 |
| Former | 24 (13.71) | 63.11±3.86 | 36.37±2.6 | 40.98±4.68 | 20.68±3.03 | 30.52±2.65 | 13.70±1.50 |
| Current | 26 (14.86) | 53.62±3.86 | 36.31±3.58 | 38.00±3.71 | 25.50±3.19 | 34.47±3.28 | 16.90±2.78 |
| *p* value ^b^ |  | 0.16 | 0.94 | 0.23 | 0.54 | 0.34 | 0.05 |
| Alcohol consumption | | | | | | | |
| Never | 125 (71.43) | 58.64±1.64 | 36.92±1.48 | 43.67±1.90 | 22.43±1.38 | 30.41±1.32 | 12.82±0.82 |
| Former | 7 (4.00) | 61.46±6.72 | 37.77±4.94 | 48.01±8.50 | 20.80±3.71 | 29.89±5.87 | 12.49±2.26 |
| Current | 43 (24.57) | 61.39±3.05 | 37.17±2.51 | 42.55±3.33 | 23.43±2.88 | 31.37±2.19 | 13.26±1.75 |
| *p* value ^b^ |  | 0.68 | 0.98 | 0.81 | 0.89 | 0.92 | 0.96 |

^a^ *p* value for Student's *t*-test; ^b^ *p* value for an ANOVA; ^#^ Significant different (*p*<0.05) by an ANOVA and Scheffe’s test.

SE, standard error.
